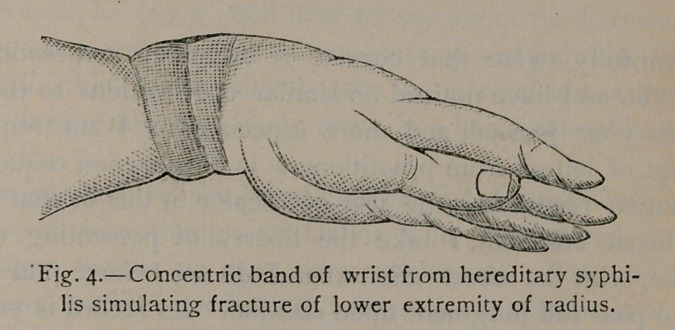# Concentric Enlargement of the Wrist in Hereditary Syphilis

**Published:** 1886-01

**Authors:** R. O. Engram

**Affiliations:** Montezuma, Ga.


### CONCENTRIC ENLARGEMENT OF THE WRIST IN HEREDITARY SYPHILIS.

BY R. O. ENGRAM, M. D., MONTEZUMA, GA.

REPORTED TO THE THIRTY-SIXTH ANNUAL SESSION OF THE MEDICAL ASSOCIATION OF GEORGIA, AT SAVANNAH, APRIL 15TH, 1885.

This enlargement of the extremities of the ulna and radius in hereditary syphilis, so far as I am informed, has not been previously commented upon by medical writers, and if it has thus far attracted the attention of the profession I am ignorant of the fact. Having had twelve cases in my own practice and the opportunity of examining a case lately under the care of Dr. J. W. Vinson, of Montezuma, Ga., I conjectured that it might be of sufficient importance to report the same to this Association.

I have not had my attention drawn a sufficient length of time to this symptom of hereditary syphilis to determine whether it is in a greater or less degree constant, or even if it is as equally so as the Hutchinson tooth found in hereditary syphilis. I believe, however, if we would examine closely, we will find it manifested quite frequently.

My cases have occurred in children ranging from one month up to one year and six months of age. Dr. Vinson's case occurred in a child about twelve months old.

The concentric enlargement has more the appearance as if two fine silk ligatures had been tied around the wrist immediately above the joint, the strands being placed about half an inch apart and tied tight enough to hide themselves in the flesh. To the touch they have all the dense hard feeling that callous does when thrown about a fracture.

I have attempted to depict somewhat the appearance of this condition by a rough sketch that is seen in figure 1 of annex cut. This was sketched off-hand from a case that fell under my care in November, 1884. The mother of this child I treated for syphilis three years previous to its birth, or rather I prescribed for a couple of Hunterian chancres. I think she received no sys-

Fig. 1.—Concentric enlargement of wrist in hereditary syphilis.

tematic treatment looking to systemic eradication. The child, when I first saw it, was covered with eruptions peculiar to this disease.

This condition often produces distortions accompanied with symptoms which are calculated to mystify the medical attendant. To illustrate, I have attempted to show in figure 2 a lateral ex-

Fig. 2.—Lateral extension produced by concentric hypertrophy of radius and ulna in hereditary syphilis.

tension of the hand of a child eight months old I now have under treatment. With the pain and swelling it might very well be mistaken for articular rheumatism. This peculiar drawn condition was due, no doubt, to excessive hypertrophy of the bone under the extensor muscles, drawing them taut.

A case fell under my care in the early part of 1884 which very much simulated fracture of the lower extremity of the radius.

This I have attempted to depict in figure 4. The similarity can be seen by examining figure 3, which represents a fracture of the lower extremity of the radius (after gross). This case was very interesting, as crepitation could be distinctly detected on

Fig. 3.—Fracture of the lower extremity of the radius (gross).

motion of the hand at the wrist-joint. This perhaps was produced from deposit or other abnormal condition of the wrist-joint. Dr. Vinson's case was similar to this, except there was no crepitation. The doctor's case might have well misled, as the child had a fall a day or so previous to falling in his charge. The Doctor was undecided as to a fracture, but with his thorough knowledge of such fractures, he was inclined to a contrary opinion.

Fig. 4.—Concentric band of wrist from hereditary syphilis simulating fracture of lower extremity of radius.

These three cases are the only ones that I have seen that could have been mistaken for other troubles.

### TREATMENT.

These cases have all been treated satisfactorily with mercury. I have used in all but three of my cases the protoiodide, in the others the bichloride, with tonics, (Huxham's tincture of bark).

One case, owing to necrosis of the ulna, necessitating operative interference, allowed me the opportunity of observing the condition of the bone, which showed great thickening of the periostium, with muscular hypertrophy of the lower extremity of the two bones of the forearm.

Dr. Vinson treated his case with inunctions of mercurial ointment rubbed in the groins and arm-pits. He informs me to-day (April 13, 1885) that it has been relieved from the trouble.

## Figures and Tables

**Fig. 1. f1:**
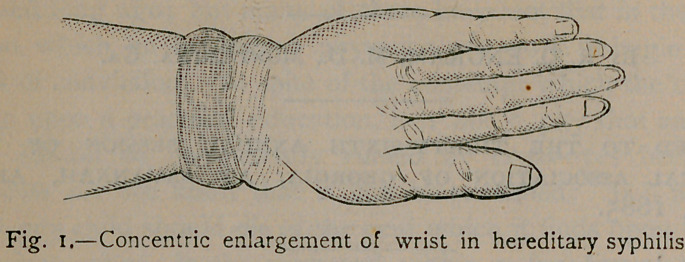


**Fig. 2. f2:**
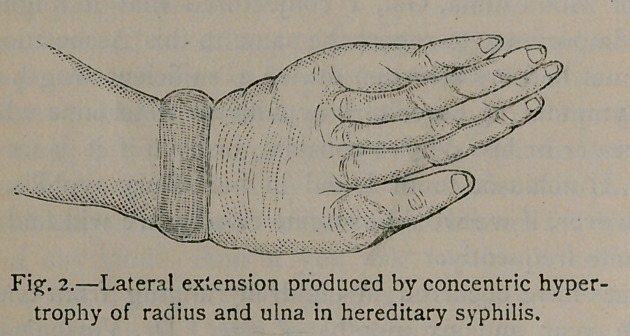


**Fig. 3. f3:**
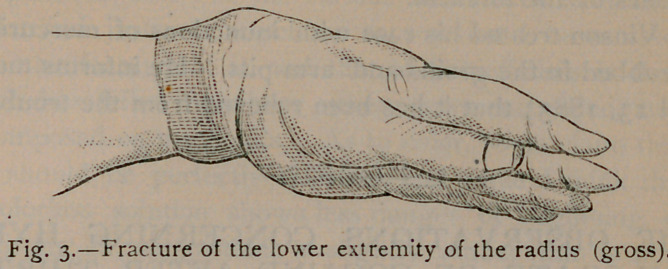


**Fig. 4. f4:**